# Clinical Characteristics and Outcomes of SMARCA4-Mutated or Deficient Malignancies: A Systematic Review of Case Reports and Series

**DOI:** 10.3390/cancers17162675

**Published:** 2025-08-16

**Authors:** Ryuichi Ohta, Natsumi Yamamoto, Kaoru Tanaka, Chiaki Sano, Hidetoshi Hayashi

**Affiliations:** 1 Department of Community Care, Unnan City Hospital, Unnan 699-1221, Shimane, Japan; nyamamoto713@gmail.com; 2 Department of Medical Oncology, Kindai University Faculty of Medicine, Osakasayama 589-8511, Osaka, Japan; katanaka@med.kindai.ac.jp (K.T.); hidet31@med.kindai.ac.jp (H.H.); 3 Community Medicine Management, Shimane University Faculty of Medicine, Izumo 693-8501, Shimane, Japan; sanochi@med.shimane-u.ac.jp

**Keywords:** SMARCA4-deficient tumor, rare cancer, immune checkpoint inhibitor, SWI/SNF complex, targeted therapy

## Abstract

**Background/Objectives:** SMARCA4-deficient or SMARCA4-mutated cancers are rare but highly aggressive tumors with poor differentiation, resistance to conventional treatments, and limited clinical guidance. While thoracic SMARCA4-deficient undifferentiated tumors are relatively well described, the full spectrum of SMARCA4-altered cancers across different organs and their therapeutic responses remains poorly understood. This study aimed to systematically review published case reports and case series to clarify the clinical characteristics, molecular features, treatment patterns, and survival outcomes of SMARCA4-altered malignancies. **Methods:** We conducted a systematic review of case reports and case series published between 2015 and 2025 using PubMed, Embase, and Web of Science. Eligible studies included adult patients with immunohistochemically or genetically confirmed SMARCA4-deficient or SMARCA4-mutated tumors. Key clinical, pathological, molecular, therapeutic, and outcome-related data were extracted. Descriptive statistics were used, and exploratory subgroup analyses were performed based on tumor type and treatment modality. The review protocol was registered in PROSPERO (CRD420251088805). **Results:** A total of 109 studies reporting 160 individual patients were included. Most tumors arose in the thorax (40.0%), followed by gastrointestinal (17.5%) and gynecologic sites (15.6%). The median age was 58 years, with a male predominance (70.0%) and frequent smoking history (44.4%). Platinum-based chemotherapy was administered in 62.5% of cases, and immune checkpoint inhibitors (ICIs) were used in 25.6%. Among ICI-treated patients, partial responses or stable disease were observed in 80.5%. The median progression-free survival (PFS) was 4.0 months, and the median overall survival (OS) was 5.0 months. **Conclusions:** SMARCA4-altered cancers are clinically and molecularly diverse but uniformly aggressive, with limited therapeutic benefit from conventional chemotherapy. Immune checkpoint inhibitors may offer improved outcomes in select patients, particularly those with thoracic tumors. Early molecular profiling, rare tumor registries, and biomarker-driven trials are crucial for guiding future treatment strategies.

## Simple Summary

SMARCA4 is a gene that plays a crucial role in regulating cell growth and development. When this gene is missing or not working correctly, it can lead to very aggressive cancers that are difficult to diagnose and treat. These cancers can occur in the lungs, digestive organs, reproductive organs, and other parts of the body, but they are rare and poorly understood. In this study, we reviewed over 100 published reports of patients with SMARCA4-deficient cancers to gain a deeper understanding of their characteristics, treatment options, and survival outcomes. We found that these tumors are usually diagnosed at an advanced stage and have a poor prognosis, although some patients benefit from immune-based treatments. By combining information from multiple cases, our study underscores the pressing need for improved diagnostic methods, molecular testing, and novel targeted therapies. This knowledge may guide doctors and researchers in improving care for patients with these rare forms of cancer.

## 1. Introduction

The SWI/SNF (switch/sucrose non-fermentable) chromatin remodeling complex plays a crucial role in regulating gene expression, cellular differentiation, and DNA repair [1]. Among its core components, SMARCA4 (SWI/SNF-related, matrix-associated, actin-dependent regulator of chromatin, subfamily A, member 4; also known as BRG1) functions as a tumor suppressor and is frequently altered in multiple malignancies [1]. Loss-of-function mutations or deficient expression of SMARCA4 have been increasingly recognized as a hallmark of a subset of highly aggressive cancers, often with poor prognosis and limited therapeutic responsiveness [2,3].

SMARCA4-deficient tumors represent a heterogeneous group of malignancies, including thoracic SMARCA4-deficient undifferentiated tumors, SMARCA4-mutated non-small cell lung cancer (non-small cell lung carcinoma), and undifferentiated carcinomas involving the gastrointestinal and gynecologic tracts [4,5,6]. These tumors are often diagnosed at an advanced stage, show resistance to conventional cytotoxic chemotherapy and immune checkpoint inhibitor-based immunotherapy, and are associated with markedly shortened overall survival [7]. Despite increasing clinical awareness, the full spectrum of SMARCA4-altered cancers remains incompletely defined, and therapeutic guidelines are lacking due to the rarity of reported cases and the absence of prospective clinical trials [7].

Although isolated case reports and small case series have provided some insights into the clinical behavior, treatment strategies, and molecular characteristics of these tumors, a comprehensive synthesis of the available evidence across tumor types remains lacking [8]. Given the rarity and clinical variability of SMARCA4-altered malignancies, systematically aggregating and analyzing published case-level data is crucial for a deeper understanding of the disease spectrum and for guiding hypothesis generation in future clinical research [9,10].

In this systematic review, we aim to collect and integrate data from published case reports and case series involving SMARCA4-mutated or SMARCA4-deficient malignancies. Our objectives are to describe the clinical characteristics, treatment modalities, therapeutic response patterns, and survival outcomes across tumor types, and to identify potential therapeutic implications. By summarizing the current evidence, we aim to clarify the clinical landscape and highlight unmet needs in the diagnosis and management of SMARCA4-altered cancers.

## 2. Materials and Methods

### 2.1. Protocol and Registration

This study was conducted as a systematic review of case reports and case series, following the PRISMA (Preferred Reporting Items for Systematic Reviews and Meta-Analyses) 2020 guidelines [11]. The study protocol was prospectively registered in the PROSPERO database (registration number: CRD420251088805). The review aimed to summarize the clinical characteristics, treatment approaches, and outcomes of patients with SMARCA4-deficient or SMARCA4-mutated malignancies. During the preparation of this manuscript, the authors utilized ChatGPT-4 (OpenAI, 2025) for language refinement, formatting assistance, and organizing preliminary drafts. The authors have reviewed and edited the output and take full responsibility for the content of this publication.

### 2.2. Search Strategy

We systematically searched PubMed, Embase, and Web of Science for English language articles published between January 2000 and June 2025. The search terms included combinations of the following: “SMARCA4” OR “BRG1”, AND “mutation” OR “deficient”, AND “cancer” OR “carcinoma” OR “sarcoma” OR “tumor” OR “neoplasm”, AND “case report” OR “case series”. The complete search strategy for each database is provided in the Supplementary Materials. Additional articles were identified through manual screening of reference lists from relevant studies.

### 2.3. Eligibility Criteria

We included studies that met the following criteria:

(1) Case reports or case series involving adult patients (≥18 years of age) with SMARCA4-deficient or SMARCA4-mutated malignancies,

(2) Confirmation of SMARCA4 alteration via immunohistochemistry or genomic testing (e.g., next-generation sequencing or Sanger sequencing),

(3) Description of at least one of the following: initial symptoms, clinical course, treatment details, treatment response, or survival outcome.

Pediatric cases were excluded to minimize clinical heterogeneity, as SMARCA4-altered malignancies in children often differ in their tumor biology, treatment approach, and prognosis. We also excluded animal studies, in vitro studies, observational cohort studies, randomized clinical trials, reviews, editorials, and studies with insufficient clinical information or unclear SMARCA4 status.

### 2.4. Data Extraction

Two reviewers (R.O. and N.Y.) independently screened all titles and abstracts, followed by a full-text review of potentially eligible articles. Discrepancies were resolved by consensus or through a third-party review (K.T., C.S., and H.H.). For each included case, we extracted the following data: publication year, country, first author, patient age and sex, smoking history, comorbidities, primary tumor site, tumor histology and stage, method of SMARCA4 detection, co-altered genes, treatment modalities, treatment responses, follow-up duration, progression-free survival (PFS), and overall survival (OS). Data were entered into a structured Excel-based extraction sheet.

### 2.5. Data Synthesis and Statistical Analysis

Descriptive statistics were used to summarize patient characteristics, tumor types, treatments, and outcomes. Categorical variables were presented as frequencies and percentages, while continuous variables were summarized using medians and ranges or interquartile ranges (IQRs), as appropriate. Progression-free survival (PFS) and overall survival (OS) were reported for cases with available data. Due to heterogeneity and the nature of the case-level data, no formal meta-analysis was conducted. Survival curves were generated using the Kaplan–Meier method for exploratory purposes. All statistical analyses were performed using EZR version 1.51 (Saitama Medical Center, Jichi Medical University, Saitama, Japan; URL: http://www.jichi.ac.jp/saitama-sct/SaitamaHP.files/OSXEN.html, accessed on 21 July 2025), a graphical user interface for R (The R Foundation, Vienna, Austria) [12]. Language editing was supported by a generative AI tool (ChatGPT 4o, OpenAI) under author supervision, strictly for grammar and style. All data extraction, analyses, and content verification were conducted manually by the research team.

### 2.6. Ethical Considerations

This study utilized only publicly available data from published case reports and did not involve any human subjects or personally identifiable information. Therefore, ethical approval and informed consent were not required.

## 3. Results

### 3.1. Study Selection

A total of 692 records were identified through database searches, including 448 from Embase, 123 from Web of Science, and 121 from PubMed. After removing 222 duplicates (219 via Covidence and three manually), 470 records remained for title and abstract screening. Following this screening, 177 articles were retrieved for full-text assessment. Of these, 68 articles were excluded for the following reasons: not an original article (*n* = 41), wrong patient population (*n* = 9), irrelevant outcomes (*n* = 8), unsuitable study design (*n* = 6), and non-English language (*n* = 4). Ultimately, 109 studies met the eligibility criteria and were included in the qualitative synthesis (Supplementary Table S1).

**Figure 1:**
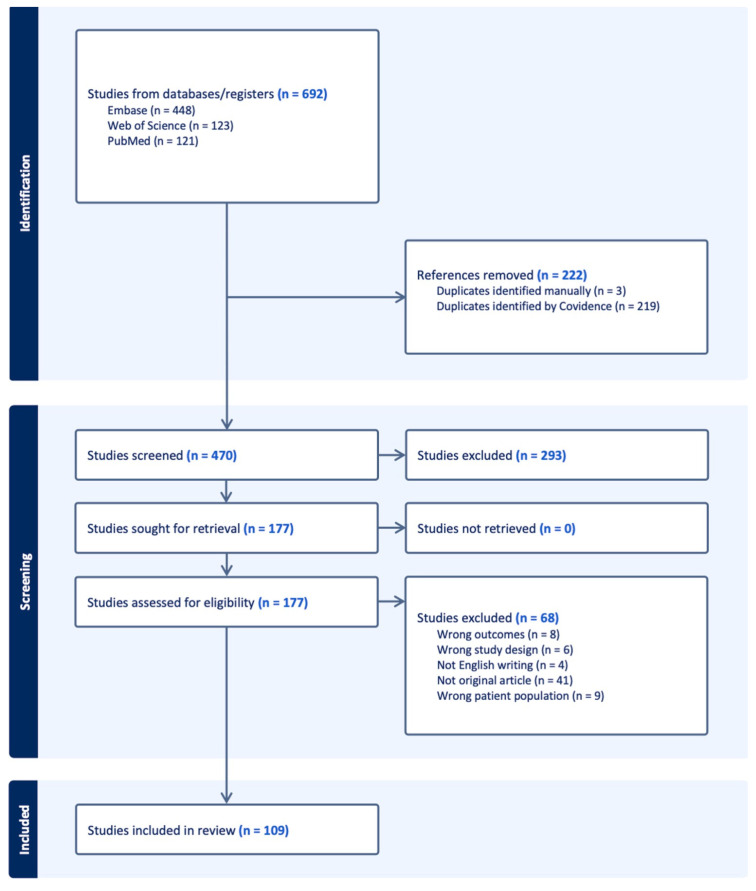
PRISMA flow diagram. Figure legend: PRISMA flow diagram of the study selection process. A total of 692 records were identified through database searches (PubMed, Embase, and Web of Science). After the removal of 222 duplicates, 470 unique records were screened based on title and abstract. Full-text articles were retrieved for 177 records, of which 68 were excluded due to ineligibility. Ultimately, 109 studies were included in the final qualitative synthesis, contributing a total of 160 individual cases of SMARCA4-mutated or SMARCA4-deficient malignancies.

After deduplication and case-level assessment, 160 individual patient cases were extracted from 109 unique publications. These included both single case reports and small case series of SMARCA4-mutated or SMARCA4-deficient malignancies, encompassing diverse tumor types, clinical presentations, and therapeutic strategies. The PRISMA flow diagram illustrating the study selection process is shown in Figure 1.

### 3.2. Study Characteristics

**Figure 2:**
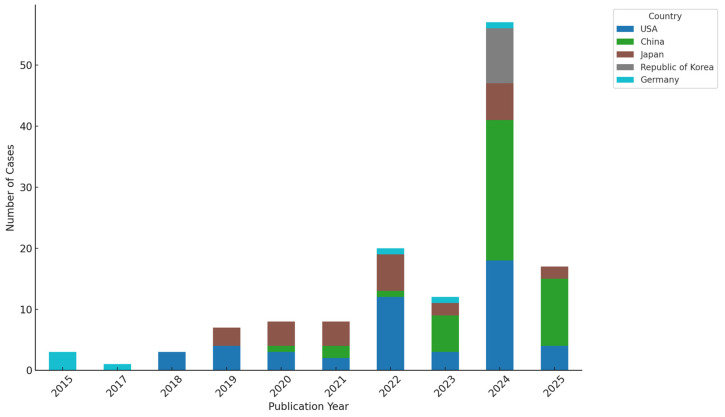
Number of reported SMARCA4-altered cancer cases by publication year and country. Figure legend: Stacked bar plot showing annual case reports from the top five contributing countries (United States, China, Japan, Republic of Korea, and Germany) between 2015 and 2025.

A total of 109 studies comprising 160 individual cases of SMARCA4-mutated or SMARCA4-deficient malignancies were included in this systematic review. The publications spanned 10 years, from 2015 to 2025, reflecting growing clinical interest in these rare and aggressive tumor types. The majority of included reports were published after 2020, with a notable rise in 2024, indicating an increasing recognition of SMARCA4 alterations in oncology. These studies originated from 17 countries, with the highest contributions from the United States (*n* = 51 cases), China (*n* = 44), and Japan (*n* = 29), followed by the Republic of Korea (*n* = 9) and Germany (*n* = 7). This global distribution highlights the widespread yet geographically varied documentation of SMARCA4-altered tumors. Most studies were single case reports, accounting for over 81% of included publications, while a smaller proportion were small case series involving two or more patients. The characteristics of these included studies are summarized in Figure 2.

### 3.3. Demographic and Clinical Features

Among the 160 patients included in this review, the median age at diagnosis was 58 years (range: 18–88), with a strong male predominance (*n* = 112, 70.0%). A history of smoking was explicitly reported or inferred in 71 of 160 patients (44.4%), while only seven patients were documented as non-smokers. Smoking history was not reported or was unclear in the remaining cases. This distribution aligns with the high proportion of thoracic tumors observed in the cohort, which are often associated with tobacco exposure.

The thorax was the most common primary tumor site (*n* = 59, 40.0%), encompassing both thoracic SMARCA4-deficient undifferentiated tumors (SMARCA4-UT) and SMARCA4-altered non-small cell lung cancers (NSCLC). Other significant sites included the gastrointestinal tract (*n* = 28, 17.5%) and gynecologic organs (*n* = 25, 15.6%). Within the gastrointestinal category, the most frequently involved organs were the esophagus (*n* = 9), stomach (*n* = 10), and colon (*n* = 3), with isolated cases involving the pancreas and small intestine. Among gynecologic tumors, the uterus (*n* = 10), ovary (*n* = 9) and cervix (*n* = 6) were the primary sites. While several studies provided precise anatomical localization, inconsistent terminology and missing details limited full organ-level classification in all cases.

Initial presenting symptoms were highly variable, reflecting the anatomical diversity of SMARCA4-altered tumors. Abdominal complaints (e.g., pain, mass, or discomfort) and general symptoms (e.g., fatigue, weight loss) were frequently reported, although documentation was absent in a subset of patients.

Tumor staging was inconsistently reported. Where described, most patients presented with advanced-stage disease (e.g., stage IV or metastatic), highlighting the aggressive nature of these malignancies. Notably, 110 patients (68.8%) had no documented metastases at diagnosis, whereas the liver (*n* = 30) and brain (*n* = 18) were the most commonly reported metastatic sites among those with advanced disease (Table 1).

When stratified by primary tumor site, brain metastases were most frequently associated with thoracic tumors, while gastrointestinal and gynecologic cancers more commonly presented with peritoneal and liver metastases. However, incomplete reporting in many case reports limited a comprehensive site-specific analysis.

### 3.4. Pathological and Molecular Characteristics

Among the 160 cases with available diagnostic information, 110 cases (68.8%) exhibited one or more features of histologically aggressive morphology: undifferentiated in 94 cases (58.8%), poorly differentiated in 11 cases (6.9%), sarcomatoid in 6 cases (3.8%), and rhabdoid in 5 cases (3.1%). Some diagnoses included multiple descriptors (e.g., “undifferentiated sarcomatoid carcinoma”), and overlapping cases were counted in all relevant categories. The remaining 50 cases (31.3%) did not include any of these four terms and were described using other classifications such as “adenocarcinoma”, “squamous cell carcinoma”, or “carcinoma not otherwise specified”.

The majority of SMARCA4 alterations were confirmed via immunohistochemistry (IHC), with descriptive terms such as “loss of SMARCA4”, “complete loss”, or “absence of BRG1 protein”. IHC-based confirmation was reported in at least 85–90% of cases, although precise numbers were variably reported. Additionally, next-generation sequencing (NGS) or other genomic assays (e.g., OncoScan) were used in approximately 43–45% of cases, often complementing IHC findings to characterize molecular profiles further.

Co-existing genomic alterations were reported in approximately 60% of cases. The most frequently co-altered genes included TP53, ALK, KRAS, FAT1, and PIK3CA. Less frequently reported alterations involved EGFR, STK11, and EP300. TP53 mutations were most common in thoracic tumors, while KRAS and PIK3CA mutations appeared more frequently in gastrointestinal tumors. However, due to heterogeneity in genomic reporting and limited sequencing in many cases, these trends should be interpreted with caution.

PD-L1 expression data were available in 33 cases, among which 28 patients (84.8%) were PD-L1-positive (≥1% expression). Four patients (12.1%) were negative, and one case (3.0%) lacked quantifiable data. These findings suggest a potentially immune-inflamed tumor microenvironment in a subset of SMARCA4-altered tumors, although testing was inconsistently performed (Table 2).

### 3.5. Treatment Patterns and Outcomes

Treatment approaches among SMARCA4-altered malignancies were notably heterogeneous, reflecting the diversity of tumor types and clinical presentations and the lack of standardized therapeutic guidance. Treatment data were available for 121 of 160 cases (75.6%), although many were described with limited or vague terminology (e.g., “chemotherapy” or “not reported”).

Among patients receiving chemotherapy, the most used agents included paclitaxel (*n* = 20), carboplatin (*n* = 18), and cisplatin (*n* = 12), often administered in combination. Other agents such as etoposide, gemcitabine, docetaxel, and nab-paclitaxel were reported less frequently. Immune checkpoint inhibitors were employed in approximately 20.6% of patients, with pembrolizumab (*n* = 15) and nivolumab (*n* = 11) being the most frequently used agents. Atezolizumab, ipilimumab, and durvalumab were also described in selected cases. Surgical interventions were reported in 42 patients, most described as tumor resection (*n* = 24) or lobectomy (*n* = 8), especially in patients with thoracic or localized disease.

Among the 160 cases with treatment response information, 61 patients (39.1%) were classified as having disease control (partial response or stable disease), while the remainder experienced progressive disease or were not evaluable. PFS data were available in 63 patients. The median PFS was 4.0 months (interquartile range (IQR), 2.0–10.5 months). OS was reported in 89 cases, with a median OS of 5.0 months (IQR, 3.0–12.0 months), which varied with treatment modalities (Table 3).

Patients who received immune checkpoint inhibitors (ICIs) demonstrated a trend toward longer PFS and OS compared to those treated with chemotherapy alone, particularly in thoracic tumors. For example, among thoracic SMARCA4-deficient undifferentiated tumors, the median OS in patients treated with ICIs exceeded 8 months, whereas those receiving platinum-based chemotherapy alone had a median OS closer to 5 months. Conversely, patients with gastrointestinal or gynecologic tumors showed less consistent responses to ICIs, and survival outcomes were generally shorter in these subgroups, regardless of therapy. Surgical resection, when feasible, was associated with improved outcomes, particularly in localized disease, although most patients presented at an advanced stage. These findings underscore the heterogeneity of SMARCA4-deficient tumors and suggest that both the anatomical origin and treatment modality significantly influence the prognosis (Table 4).

Brain metastases were reported in 28 out of 160 cases (17.5%), with the majority occurring in thoracic SMARCA4-deficient tumors. Among these 28 patients, 14 (50.0%) received radiotherapy, including whole-brain radiotherapy (WBRT) or stereotactic radiosurgery (SRS). The treatment response to radiotherapy was evaluable in 8 patients, of whom 5 (62.5%) demonstrated partial response or transient disease control. The median overall survival (OS) among patients with brain metastases was 5.5 months (interquartile range (IQR): 3.0–8.8 months), which was shorter than the OS observed in the total cohort. However, these findings should be interpreted cautiously due to potential publication bias, as cases with favorable responses may be more likely to be reported.

### 3.6. Risk of Bias Within Studies

The included studies consisted predominantly of single case reports (85.3%) and small case series (14.7%), which inherently carry a high risk of selection and reporting bias. Most reports lacked standardized outcome definitions, prospective follow-up, or comparator arms. Additionally, incomplete documentation of the clinical course, treatment rationale, and long-term outcomes was frequently observed. These limitations restrict the ability to draw causal inferences or perform formal comparative analyses. Furthermore, the variability in diagnostic methods, especially in immunohistochemical confirmation and genomic profiling of SMARCA4 alterations, adds potential measurement bias. Despite these limitations, the systematic aggregation of such real-world data remains valuable for characterizing rare malignancies and generating hypotheses for future prospective studies.

## 4. Discussion

### 4.1. Summary of Main Findings

This systematic review identified and synthesized 160 individual cases of SMARCA4-mutated or SMARCA4-deficient malignancies from 109 publications across 17 countries. The majority of these cases were reported after 2020, reflecting an increasing awareness of this distinct tumor biology. The clinical spectrum was heterogeneous, with thoracic tumors being the most common, followed by gastrointestinal and gynecologic malignancies. Most patients were male, with a high prevalence of smoking history. Initial treatments varied, with platinum-based chemotherapy and immune checkpoint inhibitors (ICIs) being the most commonly used therapies. The median progression-free survival (PFS) and overall survival (OS) were 4.0 and 5.0 months, respectively. The exploratory analysis suggested a potential survival benefit in patients treated with ICIs. Molecular profiling frequently revealed co-alterations in TP53, ALK, and KRAS. Additionally, PD-L1 expression of ≥1% was observed in 84.8% of reported cases.

### 4.2. Comparison with the Previous Literature

Prior to this review, most published data on SMARCA4-altered malignancies were limited to isolated case reports or small tumor-specific series, particularly those focusing on thoracic SMARCA4-deficient undifferentiated tumors (SMARCA4-UT) [9,13,14]. These tumors have been characterized by undifferentiated or rhabdoid morphology, frequent loss of SMARCA4 protein expression, and an aggressive clinical course [15,16]. Consistent with earlier reports, our review confirms a strong male predominance, high prevalence of smoking history, and overall poor prognosis.

However, our study expands the clinical spectrum by integrating cases across diverse anatomical sites, including gastrointestinal, gynecologic, and soft tissue tumors. This supports the evolving view that SMARCA4 alterations represent a molecularly defined oncogenic process, not restricted to thoracic origin [17,18,19]. Additionally, the use of ICIs has increased in recent years, with approximately one-quarter of patients in our cohort receiving ICI therapy [20,21,22]. Among them, roughly one-third achieved either partial response or stable disease, aligning with the emerging evidence of immunotherapy sensitivity in SMARCA4-deficient cancers. This sensitivity may be linked to a high tumor mutational burden or PD-L1 expression.

Nonetheless, comparisons with prior studies remain limited by heterogeneity in diagnostic methods, staging, and follow-up [18,23]. Most available data derive from retrospective, non-standardized reports with incomplete clinical annotation [2,24,25]. Despite these challenges, our review offers a more unified perspective on SMARCA4-altered malignancies, underscoring the importance of prospective studies, molecular stratification, and standardized reporting to enhance the understanding of treatment responses and prognoses in this rare tumor subset.

### 4.3. Clinical Implications

Our findings underscore the critical importance of identifying SMARCA4 alterations across diverse tumor types, not only in thoracic neoplasms but also in gastrointestinal, gynecologic, and soft tissue malignancies. Routine implementation of IHC for SMARCA4 and confirmatory molecular testing—such as next-generation sequencing—should be considered in diagnostically challenging or poorly differentiated tumors [26,27]. Given the aggressive clinical behavior and limited responsiveness to standard cytotoxic regimens, early integration of ICIs may offer a therapeutic advantage, particularly in tumors with elevated PD-L1 expression or high tumor mutational burden (TMB) [28,29]. Moreover, the frequent co-occurrence of TP53, STK11, and KEAP1 mutations suggests potential avenues for molecular stratification and combination therapies [28,29]. Clinicians should maintain a high index of suspicion for SMARCA4 deficiency in male smokers presenting with undifferentiated thoracic tumors but also broaden differential diagnoses to include SMARCA4-altered disease in extrapulmonary sites. Enhanced recognition and characterization of this molecular subtype may facilitate timely referral for precision oncology approaches and enrollment in future biomarker-driven trials.

Our clinical experience with thoracic SMARCA4-deficient undifferentiated tumors aligns with the trends observed in the literature, including aggressive progression, male predominance, and limited chemotherapy efficacy. Immunotherapy showed variable outcomes, underscoring the need for individualized treatment approaches.

### 4.4. Limitations

This review is subject to several limitations inherent to its reliance on retrospective case reports and small case series. The predominance of anecdotal reports introduces substantial risk of selection and reporting bias, as well as publication bias favoring unusual or treatment-responsive cases. Clinical data were frequently incomplete or inconsistently reported, particularly regarding staging, treatment response criteria, and follow-up duration. Survival metrics such as PFS and OS were inconsistently defined, and censoring data were largely unavailable, limiting the ability to generate accurate Kaplan–Meier survival curves or conduct formal comparative analyses. Additionally, while most cases included immunohistochemical confirmation of SMARCA4 loss, fewer than half underwent genomic profiling, restricting the assessment of co-mutations and genotype–phenotype relationships. The heterogeneity of tumor types, diagnostic techniques, and therapeutic approaches further complicates the interpretation and generalizability. Despite these limitations, this review represents the most comprehensive synthesis to date of SMARCA4-altered malignancies and offers valuable insights to guide future research and clinical management. Although our review found limited evidence for ICI efficacy in gynecologic SMARCA4-deficient tumors, favorable responses have been reported in specific subtypes such as small cell carcinoma of the ovaries, hypercalcemic type (SCCOHT), which often harbor germline SMARCA4 mutations and exhibit an immune-inflamed phenotype. These tumors may have been excluded from our dataset due to predefined criteria, highlighting a need for broader investigation in future analyses. Additionally, the apparent benefit of ICI in thoracic tumors observed in our dataset may reflect publication bias, as case reports with good outcomes are more likely to be published than those with no response or early progression.

### 4.5. Future Directions

To improve the clinical management of SMARCA4-deficient malignancies, prospective studies and rare tumor registries with standardized diagnostic protocols are urgently needed. These should incorporate immunohistochemistry and genomic validation of the SMARCA4 status to ensure accurate case identification. Given the observed clinical benefit of ICIs in a subset of patients, biomarker-driven trials—including basket studies targeting SMARCA4 alterations—are warranted. Furthermore, the frequent co-mutations in TP53, STK11, and KEAP1 suggest potential for combination therapeutic strategies. Beyond immunotherapy, selective inhibition of SMARCA2 (also known as BRM), the paralog of SMARCA4, has emerged as a promising synthetic lethal approach [30,31]. Recent preclinical studies have demonstrated that SMARCA4-deficient tumors are highly dependent on residual SMARCA2 function, making SMARCA2 inhibitors a rational targeted therapy option under investigation in early-phase clinical trials [30,31,32]. The integration of multi-omic profiling may further uncover actionable vulnerabilities and guide personalized treatment. International collaboration will be key to overcoming the rarity and heterogeneity of these tumors, enabling hypothesis-driven research and more effective therapeutic development.

It is also important to distinguish between tumors in which SMARCA4 inactivation is an early, disease-defining event (e.g., SCCOHT) versus those where it arises later as a secondary event during dedifferentiation. These scenarios may represent biologically distinct entities with differing genomic landscapes, morphologic transitions, and clinical behaviors. Unfortunately, most case reports did not clearly specify whether SMARCA4 loss was an initiating driver or acquired event, limiting our ability to assess this distinction. Future studies using longitudinal tumor sampling and phylogenetic analyses could help clarify the role of SMARCA4 timing in tumorigenesis and treatment responses.

While large genomic databases such as TCGA offer valuable insights into molecular landscapes, they often lack detailed clinical and treatment data for rare entities. Future research integrating public datasets with case-level clinical outcomes could enhance understanding of SMARCA4-altered malignancies.

## 5. Conclusions

This systematic review presents the most comprehensive synthesis to date of SMARCA4-mutated or SMARCA4-deficient malignancies, revealing a distinct subset of aggressive and clinically heterogeneous tumors. While limited by the retrospective nature of available data, our findings emphasize the importance of early molecular recognition, routine SMARCA4 assessment, and consideration of immune checkpoint inhibitors in appropriately selected cases. These insights provide a foundation for future biomarker-driven clinical trials and collaborative research efforts aimed at developing tailored therapeutic strategies for this rare but clinically impactful group of cancers.

## Figures and Tables

**Table 1:** Demographic and clinical characteristics.

Characteristic	Value
Age, median (range)	58 years (18–88)
Sex—male	112 (70.0%)
Sex—female	48 (30.0%)
Smoking history documented	71 (44.4%)
Most common primary tumor site	Thorax
Top 3 primary tumor sites	Thorax (59, 40.0%), gastrointestinal (28, 17.5%), Gynecologic (25, 15.6%)
Initial symptoms	Abdominal pain/discomfort, fatigue, weight loss, cough, neurologic symptoms
Cases without symptom description	9 (5.6%)
Stage at diagnosis—metastatic (stage IV or stated as such)	50 (31.3%)
Stage at diagnosis—unspecified	110 (68.7%)
Most common metastatic sites	Liver (30), brain (18), lung, lymph nodes, none
Cases without metastasis at presentation	34 (21.3%)
Cases without metastasis information	2 (1.3%)

Abbreviations: GI, gastrointestinal. (a) The most common initial symptoms were abdominal discomfort, fatigue, weight loss, cough, and neurologic symptoms. (b) “Metastatic” includes cases explicitly reported as stage IV or described as having distant metastases at diagnosis. (c) The category “most common metastatic sites” reflects sites reported in ≥5 cases; some patients had multiple sites. (d) The primary tumor site “thorax” includes both thoracic SMARCA4-deficient undifferentiated tumors and non-small cell lung cancers with SMARCA4 alterations. (e) The percentage of cases with documented smoking history was based on available data (not all cases reported this information). (f) Some categories (e.g., tumor stage, metastasis, symptoms) were inconsistently reported across studies and may not sum to 100%. (g) Metastatic patterns varied by primary site, with brain metastases more common in thoracic tumors, and peritoneal or liver metastases more frequent in gastrointestinal and gynecologic tumors.

**Table 2:** Pathological and molecular characteristics.

Category	Value
Detection method of SMARCA4 (IHC or NGS)	Reported in majority (≥86.9%) of cases
•Immunohistochemistry (IHC)-based confirmation	139 cases (86.9%)
•Next-generation sequencing (NGS)-based analysis	68 cases (42.5%)
Co-occurring genetic alterations (Top 5)	Reported in 60.6% of patients with available data
•TP53	12 cases
•ALK	5 cases
•KRAS	3 cases
•FAT1	3 cases
•PIK3CA	3 cases
PD-L1 expression (*n* = 33 cases)	33 cases reported
•PD-L1 ≥1%	28 (84.8%)
•PD-L1 <1%	4 (12.1%)
•Not quantifiable	1 (3.0%)

**Abbreviations**: IHC, immunohistochemistry; NGS, next-generation sequencing; PD-L1, programmed death-ligand 1. (a) SMARCA4 detection via IHC included terms such as “loss of SMARCA4”, “BRG1-negative”, or “complete absence of nuclear staining”. (b) Next-generation sequencing platforms included targeted gene panels, whole-exome sequencing, and hybrid capture-based assays. (c) The total number of cases with co-occurring genetic alterations was based on studies that provided molecular profiling data (*n* = 95). (d) PD-L1 positivity was defined as a tumor proportion score (TPS) ≥1%. Testing platforms and scoring systems were inconsistently reported. (e) Some studies reported qualitative or unquantified PD-L1 expression (e.g., “positive” without percentage), which were grouped as “not quantifiable”. (f) Preliminary trends suggest TP53 mutations are more common in thoracic tumors, while KRAS and PIK3CA may be enriched in gastrointestinal tumors. These observations are limited by incomplete molecular profiling.

**Table 3:** Specific treatment modalities and survival outcomes in SMARCA4-altered malignancies.

Specific Agent or Procedure	No. of Patients	Median PFS (Months)	PFS IQR	Median OS (Months)	OS IQR
Chemotherapy (*n* = 74)					
Paclitaxel	35	3.5	2.0–6	7.0	4.2–10.8
Carboplatin	34	3.5	2.2–5.8	7.0	4.0–11.5
Cisplatin	19	5.0	2.4–7.2	10.2	6.0–13.1
Etoposide	14	4.0	2.2–7.0	12.0	6.5–12.8
Docetaxel	9	2.0	2.0–2.0	4.0	3.0–5.0
Gemcitabine	9	7.0	4.5–9.5	6.0	5.0–6.0
Nab-paclitaxel	6	2.0	2.0–4.0	7.0	6.0–10.0
Irinotecan	1	NR	NR	NR	NR
Immunotherapy (*n* = 18)					
Pembrolizumab	16	4.5	3.2–5.8	6.0	4.6–9.0
Nivolumab	11	7.0	4.5–9.5	5.5	3.6–19.8
Atezolizumab	5	8.0	5.0–8.5	7.0	5.0–9.0
Ipilimumab	3	5.0	NR	14.8	9.1–20.4
Durvalumab	1	NR	NR	17.0	NR
Surgery (*n* = 42)					
Tumor resection	1	NR	NR	13	NR
Lobectomy	9	5.5	3.8–7.2	6.5	5.2–7.8
Surgery (unspecified)	38	4	2.1–5.5	6	5.0–12.0
Excision	2	NR	NR	5	NR

This table summarizes the frequency of specific chemotherapy agents, immune checkpoint inhibitors, and surgical procedures reported in the included cases, along with associated progression-free survival (PFS) and overall survival (OS) metrics. Median values and interquartile ranges (IQR) are provided for patients with available data. “NR” indicates that survival data were not reported or were insufficient for calculation. Abbreviations: PFS, progression-free survival; OS, overall survival; IQR, interquartile range; NR, not reported.

**Table 4:** Survival outcomes by tumor site and treatment modality in SMARCA4-altered malignancies.

Tumor Site	Treatment Modality	Number of Patients	Median OS (Months)	Median PFS (Months)
Thoracic	Immune Checkpoint Inhibitor	21	8.0	7.0
Thoracic	Chemotherapy	33	9.0	7.0
Thoracic	Surgery	18	9.0	4.5
Gastrointestinal	Immune Checkpoint Inhibitor	2	15.5	7.0
Gastrointestinal	Chemotherapy	2	15.5	7.0
Gastrointestinal	Surgery	12	5.5	7.0
Gynecologic	Immune Checkpoint Inhibitor	3	7.0	5.0
Gynecologic	Chemotherapy	13	7.5	3.2
Gynecologic	Surgery	8	9.5	3.2

OS: overall survival; PFS: progression-free survival; ICI: immune checkpoint inhibitor. Median OS and PFS values are approximated based on available case-level data extracted from published reports. These values should be interpreted with caution due to the heterogeneity and retrospective nature of the included data. SCCOHT and other rare gynecologic tumors with germline SMARCA4 mutations may respond to ICI therapy; such cases were not included if they lacked detailed somatic-level clinical and treatment data.

## Data Availability

The data supporting the findings of this study are available within the published literature cited in the manuscript. The data extraction table generated during the study is available in the Supplementary Materials.

## Supplementary Materials

The following supporting information can be downloaded at https://www.mdpi.com/article/10.3390/cancers17162675/s1: Supplementary Table S1. The concrete cases in the included articles.
