# Supplementary material for: Clinical Characteristics and Outcomes of SMARCA4-Mutated or Deficient Malignancies: A Systematic Review of Case Reports and Series
Source: Cancers (Basel). 2025 Aug 16;17(16):2675. doi: 10.3390/cancers17162675 (PMC12384961; doi:10.3390/cancers17162675)
Supplement: Supplementary file 1 [file cancers-17-02675-s001.zip › cancers-3795258-supplementary.pdf]

Supplementary Table 1. The concrete cases in the included articles

Year	First Author	Country	Age	Sex	Smoking History	Tumor Stage	Primary Site	Survival Outcome
2015	Abbas Agaimy	Germany	37	F	NR	Stage III	Right ovary	Alive
2015	Abbas Agaimy	Germany	34	F	NR	Stage III	Right ovary	Deceased
2015	Abbas Agaimy	Germany	34	F	NR	Stage IC	Left ovary	Deceased
2017	Abbas Agaimy	Germany	40	F	NR	cT4N2M0	Right nasal cavity and paranasal sinuses	Alive
2018	David L. Kolin	USA	34	F	NR	Advanced	Uterus	Deceased
2018	David L. Kolin	USA	33	F	NR	Full-thickness uterine invasion, ovary involvement, nodal metastases	Uterus	Deceased

2018	David L. Kolin	USA	25	F	NR	Extensive myometrial invasion (>50%), cervical, omental, adnexal involvement	Uterus	Deceased
2019	Kohichi Takada	Japan	69	F	NR	NR	Thoracic	Alive
2019	Tomoyuki Naito	Japan	43	M	Yes	T4N0M0 Stage IIIA	Left upper lobe of lung	Alive
2019	Lily Mei	USA	54	M	Yes	Metastatic	Unknown	Deceased
2019	Kei Kunimasa	Japan	45	M	Yes	NR	Thoracic	deceased
2019	Lily Mei	USA	71	M	NR	Metastatic	Unknown	Deceased
2019	Kilic, Ayse Irem	USA	70	M	NR	Metastatic	Gastroesophageal junction	Deceased
2019	Lily Mei	USA	55	F	Yes	Metastatic	Unknown	Deceased
2020	Jiahan Liu	China	40	F	No	Localized	Gastroesophageal junction	Alive
2020	Ali Kord	USA	46	F	No	Advanced	Uterus and cervix	Alive
2020	Brian D. Stewart	USA	59	M	Yes	Localized	Right upper lobe lung parenchyma	Alive

2020	Yuki Iijima	Japan	76	M	Yes	NR	Anterior mediastinum	Alive
2020	Maiko Takeda	Japan	50	M	Yes	Advanced	Left anterior mediastinum / left lung apex	Deceased
2020	Yamicia D. Connor	USA	31	F	NO	IIIB	Left ovary	Deceased
2020	Yamicia D. Connor	USA	55	F	NO	IVB	Uterus	Deceased
2020	Maiko Takeda	Japan	70	M	Yes	Advanced	Left pleura/chest wall	Deceased
2020	Yosuke Chiba	Japan	35	M	Yes	Extensive-stage disease (thoracic lymphadenopathy and bone metastases)	Right upper lobe of lung	Deceased
2020	Leckey	USA	63	M	Yes	extensive metastatic disease	Thoracic	Deceased
2021	Kei Kunimasa	Japan	51	M	Yes	Clinical stage IVA	Right upper lobe of the lung	Alive
2021	Shigeru Tanaka	Japan	58	M	Yes	Advanced	Right thorax/pleura	Alive

2021	Annie Kanchan Baa	India	62	F	No	FIGO stage IIIC	Uterus	Alive
2021	David L. Kolin	USA	29	F	NR	Advanced	Uterus	Deceased
2021	Igor Sirák	Czechia	18	F	NR	Localized	Uterine cervix	Deceased
2021	Fatma Khanchel	Tunisia	44	M	Yes	Advanced	Mediastino-pulmonary mass	Deceased
2021	David L. Kolin	USA	58	F	NR	Extensive disease with peritoneal spread	Uterus	Deceased
2021	Shotaro Ito	Japan	39	M	Yes	Advanced	Mediastinum	Deceased
2021	Nina Anžič	Slovenia	41	M	Yes	Advanced	Anterior mediastinum	Deceased
2021	Huanli Duan	China	61	M	NR	Advanced	Colon	Deceased
2021	Chen	China	46	F	NR	NR	Jejunum and ileum	Deceased
2021	Naomi Kudo	Japan	47	M	Yes	Advanced	Thoracic	Deceased
2022	Gupta	USA	48	M	Yes	Localized	Distal esophagus	Alive
2022	Takayuki Ota	Japan	59	M	Yes	NR	Stomach	Alive
2022	Shi	China	50	M	Yes	Stage IV	Left upper lobe of the lung	Alive
2022	Lan Zheng	USA	29	F	NR	T3N1M1	Uterus	Deceased

2022	Yusuke Kito	Japan	43	M	Yes	Localized	Right upper lung and anterior mediastinum	Deceased
2022	R. Grant Muller	USA	58	M	Yes	T1N1M1	Left upper lobe of the lung	Deceased
2022	Gupta	USA	70	M	No	Extensive metastasis (lung, liver) at diagnosis	Gastroesophageal junction	Deceased
2022	Gupta	USA	53	M	Yes	Localized	Distal esophagus	Deceased
2022	Omar T. Ahmed	USA	64	M	Yes	Metastatic	Distal esophagus	Deceased
2022	Omar T. Ahmed	USA	39	M	NR	Metastatic	Distal esophagus	Deceased
2022	Lan Zheng	USA	23	F	NR	T4N1M1	Uterus	Deceased
2022	Abbas Agaimy	Germany	54	M	NR	Advanced	Dorsum of foot	Deceased
2022	Takafumi Iguchi	Japan	68	M	Yes	cT2aN1M0 (Stage IIB at diagnosis)	Left upper lobe of lung	Deceased
2022	Takahiro Utsumi	Japan	72	M	Yes	NR	Right thoracic cavity (pleura)	Deceased
2022	Yoshio Nakano	Japan	73	M	Yes	Stage IVB	Right upper lobe of the lung	Deceased

2022	Yuki Meda	Japan	46	M	NR	pT3N2bM0, Stage IIIc	Rectum	Alive
2022	Ruchi Yadav	USA	76	M	Yes	NR	Right lung	NR
2022	Fulciniti, Franco	USA	69	F	NR	Localized	Left ovary	NR
2022	Gupta	USA	79	F	No	NR	Mid to lower esophagus	NR
2022	Gupta	USA	70	M	Yes	NR	Distal esophagus	NR
2023	Akriti Pokhrel	USA	36	M	Yes	Localized	Right lung pleural-based masses	Alive
2023	Jin Sheng	China	34	F	No	Advanced	Left lower lobe near posterior mediastinum	Alive
2023	Deng	China	73	M	Yes	T3N3M0, Stage IIIc	Left upper lung (lingual segment)	Alive
2023	Chihiro Takemura	Japan	42	M	Yes	Clinical T1bN1M0 Stage IIB	Right hilar / mediastinal lymph node region	Alive
2023	Pan Yang	China	51	M	Yes	T4N2M0, Stage IIIB	Right upper mediastinal mass	Alive
2023	Dominik Lobinger	Germany	50	M	NR	Advanced	Mediastinum	Alive
2023	Clemens Petrasch	Austria	68	M	Yes	Stage IV	Lung	Alive

2023	Marshall, Mason	USA	62	F	Yes	Stage IV	Right mediastinum	Alive
2023	Minoshima, Atsushi	Japan	51	M	Yes	Advanced	Thoracic	Deceased
2023	Lin, Yun-Tzu	Taiwan	69	M	Yes	Advanced	Lung	Deceased
2023	Chaoyu Pang	China	67	F	NR	Localized	Pancreas	Deceased
2023	Hee Jung Kwon	South Korea	62	M	Yes	NR	Left lung	Deceased
2023	Deborah Johanna Maartens	South Africa	48	M	Yes	IVA (T4N3M1a) – malignant pleural effusions	Anterior mediastinum	Deceased
2023	Siham Lalaoui Rachidi	USA	42	M	Yes	Metastatic	Thoracic	Deceased
2023	Ming Zhao	China	83	M	NR	cT4N0M0	Right nasal cavity and ethmoid sinus	Deceased
2023	Meng	China	65	F	NR	Stage II–III	Gallbladder	Deceased
2024	Yoko Aoyagi	Japan	28	F	No	FIGO stage IA (pT1a pN0 M0)	Right ovary	Alive

2024	Juan Lin	China	58	M	Yes	pT NR N1	Left upper lobe lung	Alive
2024	Hyeong Rok An	South Korea	61	M	NR	cT3N+M1 (IV)	Stomach	Alive
2024	Lin	China	65	M	NR	NR	Cardia of the stomach	Alive
2024	Helmink	USA	70	F	Yes	extensive mediastinal disease, osseous metastases	Mediastinal lymph nodes	Alive
2024	Morris M	USA	46	M	Yes	NR	Lung	Alive
2024	Riku Suzui	Japan	24	F	NR	Stage IB (pT1bpN0MX)	Uterus	Alive
2024	Lin	China	75	M	NR	T2N0M0	Gastric antrum	Alive
2024	Hyeong Rok An	South Korea	58	M	NR	pT4N1M0 (III)	Stomach	Alive
2024	Riku Suzui	Japan	28	F	NR	Stage IIIC	Uterus	Alive
2024	Yohei Arihara	Japan	60	M	NR	Recurrent, large retroperitoneal mass	Urinary bladder	Alive
2024	Hongji Zhu	China	64	M	Yes	Advanced	Skull base	Alive

2024	Rei Sanai	Japan	73	M	Yes	Initially stage IVA; final diagnosis: metastatic SMARCA4-UT	Lung	Alive
2024	Duan	China	56	M	No	Initial: Stage IIIA (pT3N1M0); after adrenal metastasis: Stage IV rT0N1M1b	Left upper lobe of the lung	Alive
2024	Paul F. Hanona	USA	40	M	Yes	NR	Right pleura and lung	Alive
2024	Ping Zhou	China	82	M	NR	IV	Antrum of stomach	Alive
2024	Ping Zhou	China	64	M	NR	IV	Lower esophagus and cardia	Alive
2024	Ping Zhou	China	61	M	NR	IV	Stomach	Alive
2024	Dong, Wen	China	56	M	Yes	T1bN0M0 IB	Left lower lobe of lung	Alive
2024	Kensuke Takei	Japan	40	M	Yes	cT3N2M0	Left upper lobe of the lung and adjacent chest wall	Deceased

2024	Kensuke Takei	Japan	74	M	Yes	cT4N0M0	Left upper lobe and adjacent 3rd–5th ribs	Alive
2024	Yin	China	63	M	Yes	Stage IIB	Right upper lobe of the lung	Alive
2024	Yu Qi	China	77	F	NR	Confined to cervix	Cervix	Alive
2024	Gumusgoz	USA	67	M	No	Localized	Skin	Alive
2024	Petar Popov	Austria	43	F	Yes	pT3, pNx, L0, V0, R0	Mediastinum / thorax	Alive
2024	Morris M	USA	47	M	Yes	NR	Brain	Deceased
2024	Samuel MacDowell	USA	40	M	No	Likely Stage IV	Right upper lung apex	Deceased
2024	Hongji Zhu	China	34	M	Yes	Advanced	Left nasal cavity	Deceased
2024	Morris M	USA	67	M	No	NR	Lung	Deceased
2024	Morris M	USA	36	M	Yes	NR	Unknown	Deceased
2024	Hyeong Rok An	South Korea	64	M	NR	cT4N3M0/1 (III/IV)	Stomach	Deceased
2024	Hyeong Rok An	South Korea	56	M	NR	cT2/3N0M1 (IV)	Gastric antrum, anterior wall	Deceased
2024	Hyeong Rok An	South Korea	67	M	NR	cT3/4N+M1 (IV)	Stomach	Deceased
2024	Faris Shweikeh	USA	88	M	NR	IV (metastatic)	Distal esophagus	Deceased

2024	Hyeong Rok An	South Korea	21	M	NR	cT3/4N3M1 (IV)	Stomach	Deceased
2024	Faris Shweikeh	USA	53	M	Yes	IV (metastatic)	Distal esophagus	Deceased
2024	Morris M	USA	66	M	Yes	NR	Lung	Deceased
2024	Morris M	USA	58	M	Yes	NR	Lung	Deceased
2024	Chandan C S	India	30	F	NR	Advanced	Ovary	Deceased
2024	Faris Shweikeh	USA	49	F	No	IV (metastatic)	GE junction/distal esophagus	Deceased
2024	Helmink	USA	58	M	Yes	extensive disease with bone and nodal involvement	Iliac bone mass	Deceased
2024	Amanda Craven	USA	73	M	NR	Metastatic	Unknown	Deceased
2024	Ming Zhao	China	61	F	NR	cT4N0M0	Left nasal cavity and ethmoid sinus	Deceased
2024	Yuanhang Wang	China	55	M	NR	Stage IV	Right pleura/lung region	Deceased
2024	Wei, Xiaoling	China	69	M	Yes	Stage IV	Thoracic	Deceased
2024	Shuai Luo	China	67	M	yes	Metastatic	Presumed lung primary	Deceased

2024	Di Yang	China	51	M	Yes	NR	Posterior mediastinum	Deceased
2024	Shui Ho Chan	USA	55	M	Yes	Clinical Stage IVB	Left upper lobe of lung	Deceased
2024	Yi-Nan Shi	China	43	F	No	Clinical stage IV	Duodenal papilla	Deceased
2024	Yi-Nan Shi	China	51	M	No	Clinical stage IV	Duodenal ampulla	Deceased
2024	Ping Zhou	China	61	M	NR	IV	Lateral posterior wall of stomach	NR
2024	Helmink	USA	70	M	Yes	NR	Right paratracheal mass	NR
2024	Weiping Dai	China	79	F	NR	Stage IVB	Right middle lung lobe	NR
2024	Oestreicher, David	Germany	81	F	NR	Localized	Left parotid gland	NR
2024	Hyeon Gyu Kang	Republic of Korea	72	M	NR	cT4N3M0	NR	NR
2024	Akira Kambe	Japan	76	M	Yes	Metastatic	Unknown	NR
2024	Hyeon Gyu Kang	Republic of Korea	76	M	NR	cT3aN0M0	NR	NR
2024	Hyeon Gyu Kang	Republic of Korea	68	M	NR	cT4aN0M0	NR	NR
2024	Hyeon Gyu Kang	Republic of Korea	52	M	NR	cT4N2M1	NR	NR

2024	Hyeon Gyu Kang	Republic of Korea	74	M	NR	cT4N2M0/1	NR	NR
2024	Hyeon Gyu Kang	Republic of Korea	86	M	NR	cT4bN0M0	NR	NR
2024	Lin Chen	China	50	F	NR	Advanced	Stomach	NR
2024	Hyeon Gyu Kang	Republic of Korea	47	F	NR	pT4bNxM0	NR	NR
2024	Hyeon Gyu Kang	Republic of Korea	77	M	NR	pT2N0M0	NR	NR
2024	Hyeon Gyu Kang	Republic of Korea	50	M	NR	pTxN2bM0	NR	NR
2024	Chandan C S	India	40	F	NR	Localized	Ovary	NR
2024	Deng Xiaoxu	China	65	F	NR	pT2a pN2 pM0, Stage IVB	Gallbladder	NR
2024	Morris M	USA	68	F	NR	NR	Lung	NR
2024	Manasi Mundada	India	55	M	Yes	NR	Right upper lobe lung mass	NR
2025	Yang	China	64	F	NR	NR	Endometrium	Alive
2025	Lu Wang	China	68	M	Yes	Metastatic	Lung	Alive
2025	Dong, Jie	China	67	M	Yes	Advanced	Left lower lobe of lung	Alive
2025	Mariano Lombardi	Italy	63	F	Yes	Localized	Right lower lobe of lung	Alive

2025	Pan	China	54	M	No	Stage IV	Left upper lobe of the lung	Alive
2025	Yokoe, Takuya	Japan	40	F	NR	FIGO stage IB1 (clinical T1bN0M0)	Uterine cervix	Alive
2025	Yuanli Zhong	China	76	M	NR	NR	Stomach	Alive
2025	Yuanli Zhong	China	72	M	NR	pT4aN3bM0	Stomach	Alive
2025	Junmin Song	USA	54	M	Yes	NR	Anterior mediastinum	Alive
2025	Rong Xiao	China	59	M	Yes	Metastatic	Lung	Alive
2025	Liu	China	71	M	Yes	Stage IV	Right lower lobe lung mass	Alive
2025	Xiaotong Qiu	China	53	F	No	cT2bN3M1c (Stage IVB)	Right lower lobe of lung	Deceased
2025	Yuanli Zhong	China	57	M	NR	cT4aN3aM1	Stomach	Deceased
2025	Thomas Harrington	USA	78	M	Yes	Advanced	Right suprahilar/mediastinal region	Deceased
2025	Elaina Daniels	USA	41	F	NR	Advanced	Uterus	Deceased
2025	Sunayana Misra	India	28	M	No	ypT3 pN1	Gastric cardia	Deceased

2025	Nada Shaker	USA	69	M	Yes	Advanced	Lung	Deceased
2025	Yukina Izumi	Japan	44	M	Yes	IVB	Mediastinum	Deceased
2025	Yuanli Zhong	China	73	M	NR	pT4bN1M1	Stomach	Deceased

Footnote

NR – Not Reported: Information not provided in the source material. FIGO – International Federation of Gynecology and Obstetrics: Staging system commonly used for gynecological cancers (e.g., uterine, ovarian). cT / cN / cM – Clinical Tumor / Node / Metastasis Staging: Based on clinical assessment (e.g., imaging, physical exam). cT: Clinical tumor size/extent. cN: Clinical regional lymph node involvement. cM: Clinical distant metastasis. pT / pN / pM – Pathological Tumor / Node / Metastasis Staging: Based on histopathological examination after surgery. pT: Pathologic primary tumor extent. pN: Pathologic regional lymph node involvement. pM: Pathologic distant metastasis. Stage IVB – Advanced Cancer Stage: Distant metastases, typically inoperable or widely spread disease. Extensive-stage disease – Term often used in small-cell lung cancer to indicate disease spread beyond one hemithorax or distant metastasis. GE junction – Gastroesophageal Junction: Area where the esophagus meets the stomach. Metastatic – Cancer that has spread from the primary site to distant organs. Localized – Cancer confined to the primary site without regional or distant spread. Alive – Patient reported as living at the time of case report or follow-up. Deceased – Patient reported as having died at the time of the case report or follow-up. Thoracic – Refers to the chest area; may include the lung, mediastinum, or pleura. Anterior Mediastinum – The front compartment of the mediastinum, often the site of thymic tumors. Advanced – Often used when staging details are incomplete but suggest progression beyond early stages. Suprahilar/mediastinal region – Area above the hilum of the lung, including central lymph nodes and vessels.
